# Diphtheria and Its Treatment, Based on Recent Investigations

**Published:** 1883-11

**Authors:** J. M. Keating

**Affiliations:** One of the Attending Obstetricians to the Philadelphia Hospital, and Lecturer on the Diseases of Women and Children, etc.


DIPHTHERIA AND ITS TREATMENT, BASED ON RECENT INVESTIGATIONS. A Clinical Lecture delivered at the Philadelphia Hospital December 6, 1882. By J. M. KEATING, M.D., one of the Attending Obstetricians to the Philadelphia Hospital, and Lecturer on the Diseases of Women and Children, etc. Reported by WILLIAM H. MORRISON, M.D.

At the last lecture, as you may remember, I spoke of some of the acute varieties of diseases of the throat in children. I propose to-day to make some remarks in reference to the clinical aspect of the subject of diphtheria, based upon the experiments upon observations in the practice of some of us who are interested in this subject at the present time. At my last lecture, I pointed out to you the differential diagnosis of the various forms of acute laryngeal disease in children; these were spasmodic laryngitis, or false croup, one of the most important and most frequent of these diseases; a combination, as it were, of simple acute laryngitis, and laryngismus stridulus, and pseudo-membranous laryngitis, or true croup. I showed you at that time how pseudo-membranous laryngitis differed in its symptoms from spasmodic laryngitis, especially in this fact, that in the former there was present a false membrane, which, by causing mechanical obstruction and interfering with respiration, gives rise to other symptoms not found in the latter disease. At the present time the most important subject which can come before us is the difference, if there be any, between pseudo-membranous laryngitis and the disease known as diphtheria, which is also characterized by a membranous deposit upon the mucous membrane of the throat, fauces and nares, sometimes extending into the larynx and trachea, giving rise to various symptoms, many of which are due to the presence of membrane obstructing the tracheal opening. I believe that the former affection is always associated with the laryngeal and tracheal forms of the latter, usually presenting its most marked symptom, but that it can occur by itself as the result of cold or irritation as well as its analogue croupous pneumonia. I told you that the mucous membrane of the pharynx differs anatomically from that of the larynx and trachea, and that, therefore, irritation and inflammation of these two parts give rise to entirely different sets of symptoms. The mucous membrane of the pharynx is covered by a thick, almost impenetrable layer of squamous epithelium. This is placed on a loosely-attached submucous tissue which is capable of being greatly distended by infiltration and by blood, so that inflammation of this membrane will cause congestion and œdema to take place below the epithelium, and may give rise, at the same time, to the appearance of but slight congestion of its surface when the disturbance beneath is very great. The mucous membrane of the larynx and trachea is covered by the ciliated form of columnar epithelium. This epithelium is placed upon a very dense basement membrane, and has behind it thick inelastic cartilage. Inflammation at this point will be rapidly followed by the appearance of fibro-plastic lymph upon the surface, lifting the easily detached epithelium, and giving rise to layers of membrane ; these deposits may form rapidly, and quickly occlude the larynx or trachea.

About the year 1868, Oertel found in the blood of persons suffering from diphtheria, a form of micrococcus, a peculiar granular matter. A full account of the subject will be found in Ziemssen's *Encyclopedia*, and in the supplement of the report of the National Board of Health for last year, but as this latter is not accessible to many of you, I go into this subject somewhat in detail. Many observers subsequently found that this peculiar matter, which occasionally took the form of little black granules and again appeared in masses, was present in all cases of diphtheria, and in proportion to the severity of the attack. The presence of these micrococci has been positively substantiated, not only in diphtheria, but also in almost all of the so called zymotic affections. Those of you who attended the lectures in this hospital last spring, will remember the severe epidemic of measles in the children's ward at that time. The children died very rapidly, and it was impossible at first to account for their rapid death. Dr. Formad and myself made a number of observations on the blood of these cases, and it was found that as soon as the malignant symptoms presented themselves, the blood of these children was filled with micrococci. There were immense numbers of these; not only did they float in the liquid in masses, looking like frog spawn on a pond, but they also penetrated the white corpuscles, and increased in numbers to such an extent as to cause the globule to burst in many cases, although the red corpuscles were not attacked. The severity of the symptoms was found to be in proportion to the number of micrococci. Whether this was due to the micrococci, or whether the severity of the symptoms and the presence of the micrococci were simply coincident, I am not prepared to positively assert; we do not know, however, that where this material was found in the blood, those children were in danger of death from the malignancy of the disease and from exhaustion, and also from heart clot. It has been found by many observers that these micrococci exist not only in the part where they are introduced, as in the blood of the throat in diphtheria, but they are also carried to the finest capillaries in the body, multiplying with great rapidity. They have been found in the marrow of the bones, and in the minute vessels of the kidney, clogging them, causing mechanical congestion, and finally, either causing or attending a distinct diphtheritic process in the kidney analogous to that going on at the point of invasion. We observed in this epidemic of measles that the children would often exhibit extraordinary symptoms connected with the respiration. A child would be doing well, when suddenly it would seem to have difficulty in breathing. There would probably be, as is usual in such cases, a certain amount of pulmonary congestion and some catarrhal pneumonia, but at the same time the severity of the pulmonary lesions would not be in proportion to the disturbance of respiration which was present. The gasping spells were shown to be due to the fact that insufficient blood passed through the lungs to be oxygenated. There was no trouble about getting air into the lungs, but the trouble was to get venous blood from the right ventricle to travel into the lung.

There was some obstruction. After death it was found to be due to a white clot, which had evidently formed sometime before, and upon microscopical examination this clot would be found to be filled with micrococci.

This is an extremely interesting subject, and those who have studied these conditions under the microscope have found that it was not only an interesting subject in itself, but one of great practical importance. I wish to-day, in view of the present epidemic, to speak to you of some of the practical points which this matter brings before us. It is also of great importance in connection with treatment! From the experiments of Doctors Wood and Formad—experiments which have been verified and which are still going on—it has been proven that the initial lesion in cases of diphtheria is in the throat. It has for many years been a question whether diphtheria was like typhoid fever, scarlet fever and other affections where the blood was first affected by the poison of the disease and a period of incubation resulted, and that this was finally followed by the peculiar characteristics of the disease, that is to say, the appearance of membrane in the throat in diphtheria, the scarlatinous rash in scarlet fever, and of rose colored spots and so on in typhoid fever. These experiments have, I think, conclusively shown that diphtheria is a contagious disease, contagious from the membrane which is present, and that this contagious principle can be inoculated, can be transmitted directly to the individual, and also that it first appears upon that part where the inoculation has been performed independent of any lengthy period of incubation. If the membrane from a case of diphtheria is inoculated upon any part, it is at this point where the first manifestations will occur, which the constitutional disturbance will be of a secondary character. In all cases of diphtheria examination of the blood under the microscope shows the presence of these micrococci, and also that they first appear at the point of greatest intensity, or otherwise invasion, and thence make their way through the circulation to other parts of the body. In the primary stage they are found in the membrane and the epithelium on the surface of the tongue, mouth, and throat, and from these parts they rapidly penetrate into the submucous tissue, and finally are taken up by the bloodvessels and lymphatics, and develop with great intensity. These experimenters have declared that their observations have shown that diphtheria need not be a so-called specific affection ; that in rabbits, any inflammation of the mucous membrane of the throat may produce symptoms analogous to those of diphtheria with the presence of microrocci ; they at the same time tell us that these micrococci are always found to a greater or less extent upon the mucous membrane of the throat and epithelial débris on the tongue of those in health, but not necessarily in the air which we breathe, unless it be foul. I can scarcely see my way clear to go as far as this, but believe that in a dormant state they may have their peculiar home in the epithelial detritus which is found in the mouth, and are deposited there and vivified by foul air from cesspool gases, and then if any irritation or denudation of the mucous membrane takes place from cold, from sore throat of any kind, especially in children, in whom there is a special vulnerability, these micrococci suddenly develop with great intensity and rapidly produce the malignancy which we see in the secondary manifestations of the diseases ; their habitat being foul air, whence they are derived.

Let me here refer to the great error that is frequently made of calling one disease after another, *e. g.*, calling a sore throat *diphtheritic* because it has a membrane upon it, or a disease *typhoid* because it resembles typhus, or a rash *scarlatinous* because it resembles that of scarlet fever. The result of this is that people who do not know what these terms mean imagine that they have these various diseases, and you will be often told that a person has diphtheria every fall, because some one has called it diphtheritic sore throat, or that a child has had scarlet fever a dozen times, when in reality it has suffered from attacks of erythema, or a person has been *threatened* with a disease of this sort ! This use of terms is objectionable to those who are trying to attain a scientific accuracy in regard to the diagnosis and treatment of these diseases, and raises doubts in the popular mind.

Diphtheria, though possibly a disease which may occur more than once, carries with it a certain individuality of its own. The poison, whether it be micrococci or not, requires something which will increase its activity, and will, under ordinary circumstances, lie dormant. It may remain for some time in a pure atmosphere, and not develop the disease ; but if it is exposed to sewer-gas or bad ventilation, this growth, this cause of diphtheria, is vivified, producing usually the primary affection of the throat, and eventually involving the general system ; or the contagion may be direct from one individual to another. Experiments have proved this, in the fact that the most virulent of micrococci, that of erysipelas, if exposed to air which is pure, or, in other words, to oxygen, loses its intensity, and when inoculated shows its enfeebled powers.

Is this disease identical with true croup or pseudo-membranous laryngitis? In certain cases it may be Oertel, in Ziemssen's *Encyclopedia*, describes two forms of the disease—the catarrhal and the croupous. The first is the result of catarrhal inflammation, which he supposes to be due to the action of a small amount of poison on the throat membrane, giving rise to a mild form of catarrh. This catarrh may come from any irritation. It is no more due to the specific action of diphtheria poison than it is due to the inhalation of fumes of nitric acid or of any other irritating substance. It is simply a superficial catarrh similar to a catarrh in the head. Thus, if you inhale the dust of ipecac, you will set up as severe a catarrh as if you go out into the night air without your hat. Then again, we have that which is described as the croupous form, which may also be dependent upon a specific blood poison, and has attending it a fibrinous exudation, with rapid failure of the system following the introduction of the poison, producing adynamia. We know that ordinary causes, presumably not dependent on diphtheretic poison, may produce laryngitis with the deposit of membrane. Children with this disease present certain appearances and symptoms which are not like those that are seen in the early stages of diphtheria. Those of you who have seen the two affections side by side will, I think, agree with me in saying that they are not identical in appearance. In a case of pseudo-membranous laryngitis, the child may have deposits in the trachea which may so obstruct the tracheal opening as to cause great interference with respiration, yet until the fatal signs come on the symptoms are not identical with those of diphtheria ; there is not that profound prostration, that swelling about the neck and angle of the jaw which shows great involvement of the lymphatics, with enlargement of the glands. In the former disease, the lymphatics are not at all affected in the early stage ; and, again, when I tell you that more or less membranous deposit may be readily caused by the least irritation, you can easily see that it is not necessary to always account for its presence by a violent poison acting upon its surface. In the most severe forms of diphtheria the disease, originating in the pharynx, has a tendency to extend into the nasal cavity. In some cases, in adults, the larynx escapes almost entirely, or again, the primary lesion may be in the trachea. Diphtheria is not limited to young children, but is quite common between the ages of ten and fifteen years, and is as fatal at these ages as it is at the same number of months. Pseudo-membranous laryngitis, on the other hand, is quite rare after the age of five years.

It was not my intention to day to point out these differences. There are some authorities who believe that the two diseases are identical, while others take the opposite view. For my own part, I take a middle course. I think that diphtheria may cause croup, as may any other irritant ; they both may be present in the same case, and probably are in children, and one may be the exciting cause of the other. There is in children a tendency for all catarrhal conditions of the mucous membrane of the air passages to exist, and I think we may look upon pseudo-membranous laryngitis as a constant symptom of diphtheria at this age ; still, we know that diphtheria can occur in adults without pseudo-membranous laryngitis being present, and that the latter can take place in children with abundant deposits, but unaccompanied by lympathic involvement and adynamia, unless septicæmia should set in from putridity of the false membrane.

My purpose to-day is to make some remarks in connection with treatment. In treating those cases of measles to which I have alluded, we used large doses of alcohol, in the form of whisky, and we found that it diminished the intensity of the malignant symptoms, and, at the same time, lessened the number of micrococci in the blood. We examined the blood and determined the number of micrococci present ; the child was then placed upon full doses of whisky, and examination of the blood then showed that the number of the micrococci had sensibly diminished, and that they seemed paralyzed in their development. They did not have that tendency to duplication which they had before shown. These investigations were continued by Mr. Oliver Hopkinson, at my suggestion, and he has obtained some extremely interesting results. I might as well state here that we are not able to distinguish under the microscope the different varieties of micrococci, as they present exactly the same appearance; still we may say that the micrococcus of diphtheria may differ from that of erysipelas or of measles, but that though the virulence differs in each case, they are all affected, more or less, in their development by the administration of this same drug. Dr. J. G. Richardson very happily illustrated this point before the Philadelphia County Medical Society. He said that although these growths appeared exactly the same under the microscope, yet they could not be considered on that account as being the same; the spermatozoa of a colored man and those of a white man may look exactly alike under the microscope, yet we know that the spermatozoa of one would not produce the other, and we can illustrate it further by adding that they would succumb alike to the same toxic agents.

From the experiments to which I have alluded, we may conclude that anything that will kill or retard the growth of these micrococci has the effect either of so paralyzing them as to prevent them from producing the malignant symptoms, or else of placing the blood of the individual in such a condition as to prevent the growth of the micrococcus. We find that the fatality of the disease accords with the presence of micrococci, and that if we can destroy the latter, we can diminish the former. As I have already said, alcohol has been found to have a curative effect. The same thing has been observed in regard to corrosive sublimate. The effect of this latter remedy was marked in the experiments of Mr. Hopkinson. He also found that chlorine and chlorine compounds exercised an even more pronounced effect. These experiments are very important. It has been known for many years that the various compounds of chlorine, as chlorate of potassium, tincture of the chloride of iron, chloride of sodium, corrosive chloride of mercury, calomel and soda, chloride of lime, hydrochloric acid, and many others, had a curative or antidotal effect in these malignant conditions, or in what has been termed septicæmia. I believe that in all cases of this kind we have two different conditions; one is this tendency to malignancy accompanying the increased growth of these micrococci, which may be prevented by the use of alcohol, chlorine, and the like; the other is a septicæmia, associated with the other, resulting from a condition which is entirely different from the former, and where we have under the microscope micrococci and rod-like bacteria, due to decomposition and putridity. These latter produce symptoms of septicæmia, which are associated with those produced by the specific poison. We know that carbolic acid prevents the development of the rod-like bacteria, but it has been stated that this drug has no effect on the micrococcus. We find, by experiments on animals, that if a certain number be poisoned with the poison of erysipelas, diphtheria, they will die; while if others, poisoned in the same way, at the same time receive injections of chlorine water, they will live; and when we find that these experiments are made day after day with the same result, the logical conclusion to which we arrive at present is that, to some extent—how great we can not now say—chlorine and its compounds are antidotal to these diseases.

To review this subject we have noted that micrococci are associated with diphtheria, that inoculation of these matters will produce the disease, that they are found at that point where the diphtheritic process is found in the greatest intensity, and lastly that certain solutions will kill or paralyze these micrococci, and prevent their growth. Let us now study a form of treatment based upon these views. We divide our treatment into the constitutional and the local. For the former we rely on hourly administration of liquid food in small quantities, such as beef-juice, wine-whey, milk, frozen beef-tea, or ice cream, albumen water, or peptonized food by the bowel; also stimulants, fresh vegetable acids, koumiss; and drugs, as quinine, strychnine, etc. We must pay a special attention to the circulation, as *heart death* may occur in two ways: First, syncope from fatty degeneration of the cardiac muscle; and sudden death, occurring even during convalescence. This requires absolute quiet in bed, digitalis, and strychnine. Second, heart clot, which is most insiduous in its formation, and which, I believe, may be due to the rapidly accumulating micrococci, which can possibly be prevented by free stimulation, chlorine, and the chlorides, both internally and by inhalation, and *fresh air*. The local treatment has three objects in view—namely, the destruction of the diphtheritic poison or germ; the prevention of putridity of the false membrane, and the allaying of the inflammation of the mucous membrane itself. It is necessary for the latter indication to keep the air moist; either by the slaking of lime, to which may be added the vapor of chloride of lime; vaporizing the throat by means of an atomizer, to which may be added opium or hyoscyamus in some form, and applying moisture to the throat without, either with a wet cravat covered with oil silk or a light poultice surrounding the neck. For the treatment of diphtheria I would recommend astringent local applications, as, for instance, the perchloride of iron, believing that the disease is originally a local affection. I would not waste time in using carbolic acid alone, for I believe that in such cases, at the onset, it is of questionable use. An excellent local application, which should be used daily by the physician, applied thoroughly to the whole mucous membrane of the fauces. is:

℞.—Tinct. iodin.......................... f℈j.

Acid. carbol.......................... gr. x.

Tinct. ferri perchlor................ f℈j.

Glycerinæ ............................ad. f℥j.

The use of the carbolic acid being to prevent decomposition of the membrane. Between times I have used a gargle of salicylic acid and claret. Common salt, I have no doubt, would be of great service, and probably the popular opinion so favorable to chloride of sodium and sulphur will be found to be based upon a very solid foundation. You know that calomel and soda, in small and frequently repeated doses, placed dry upon the tongue, has many upholders, and probably its local action is a very important one, as much so as the constitutional one in rendering the membrane aplastic. Labarraque's solution would be certainly indicated, and possibly the very valuable Platts' chlorides would serve the same good purpose.

But remember the importance of early local treatment with strong astringent applications, in proportion to the severity of the attack. The presence of false membrane will be followed by *two* evil results: Mechanical obstruction, which will cause death, and its decomposition, which will produce septicæmia. These are both totally independent of the disease itself, and may be classed as complications, though in reality they are taken as symptoms.

Early tracheotomy may, but most frequently does not, prevent the former; quinine, iron, and food, with local antiseptics, may ward off the latter, provided the hidden and unapproachable recesses of the nasal cavities do not render local treatment impossible, and constitutional supporting treatment unavailing. I would then give brandy in gradually increasing doses, giving it in proportion to the age of the patient and his condition, and at the same time administer chlorine in some of its forms, as tincture of the chloride of iron, or chlorine water (f℥j or less, three or four times dally, and increasing dose), which has been advocated for a long time by Dr. J. L. Ludlow, of this city, and which has been used by him with great success. I would use the vapor of chlorine in the room.

℞.—Sodium chloride........................ 7½ parts.
Bin-oxide of manganese............... 6 "
Add strong sulphuric acid............q. s. (Edis.)

Dr. William Pepper recently reported a case of very severe diphtheria cured by the use of large doses of corrosive sublimate. Chlorate of sodium has been used with success by Dr. Ludlow, and is more soluble than the chlorate of potassium, and therefore more quickly enters the system. It can be given in eight or ten-grain doses every three or four hours. Here you see that the experiments in the laboratory have a practical value. They give us something tangible to work on. We find that chlorine does good; we use it and its compounds locally, freely, at first upon the mucous membrane itself, because we know that this is the initial point at which the disease originates. We try to kill or paralyze this micrococcus before it enters the circulation, for after it passes into the blood it is difficult to influence it. A curious confirmation in regard to this is found in the article by Prof. Oertel. He suggests that when the throat is very much involved, when the swelling in the connective tissue is great, when there is marked stiffness of the muscles of deglutition, and great redness and the appearance of membrane on the throat, a hot poultice should be applied to the outside, for the purpose of encouraging suppuration, believing that by increasing the afflux of blood to the submucous tissue of the throat, and increasing in that way the tendency to suppuration, there is a barrier erected to throw off this poison and prevent its absorption. We cause a layer of pus to develop, and the entrance of these micrococci can be then prevented.

If the poison of diphtheria, the micrococcus of that disease, is found constantly with the epithelial detritus upon the tongue and throat and other mucous membranes, and needs but foul air, absence of oxygen, debilitated constitution, and an abrasion to awaken it from its downcast condition, why can not auto-infection take place in many cases apart from exposure to a contagium of the same kind from without? Could not membranous deposit from simple catarrh, or pseudo-membranous laryngitis with decomposition of the membrane be the exciting cause, and diphtheria be engrafted by an exciting cause from *within* as well as by bad sewerage from a faulty closet, or infected drinking water? I believe that septic or decomposing matters will rapidly vitalize the diphtheritic micrococcus in the throat and fauces, as well as in the uterus and vagina, and I also believe that apart from that, the micrococcus will develop symptoms of malignancy independent of septicæmia alone. In the former iron, quinine, alcohol, and chlorine compounds should be used largely internally, and carbolic acid as an application, with a free supply of oxygen and nourishment; in the latter cases, alcohol, nourishment, fresh air, the chlorine compounds, and vegetable acids are your sheet-anchor—with local astringents.

Although these points which I have brought before you to-day are the results of theory, I should not have presented them if they had not been confirmed by practice. We know that these various compounds of chlorine have been used with good results, and we simply endeavor to connect the theory and the practice, to show why these remedies have proven of benefit.—*Medical News.*

